# Complement Components C3 and C4 Indicate Vasculitis Manifestations to Distinct Renal Compartments in ANCA-Associated Glomerulonephritis

**DOI:** 10.3390/ijms22126588

**Published:** 2021-06-19

**Authors:** Samy Hakroush, Désirée Tampe, Peter Korsten, Philipp Ströbel, Björn Tampe

**Affiliations:** 1 Institute of Pathology, University Medical Center Göttingen, 37075 Göttingen, Germany; samy.hakroush@med.uni-goettingen.de (S.H.); philipp.stroebel@med.uni-goettingen.de (P.S.); 2 Department of Nephrology and Rheumatology, University Medical Center Göttingen, 37075 Göttingen, Germany; desiree.tampe@med.uni-goettingen.de (D.T.); peter.korsten@med.uni-goettingen.de (P.K.)

**Keywords:** acute kidney injury, innate immunity, complement system activation, autoimmune diseases, systemic vasculitis, ANCA-associated glomerulonephritis

## Abstract

Acute kidney injury (AKI) is a common and severe complication of antineutrophil cytoplasmic antibodies (ANCA)-associated vasculitis (AAV) causing progressive chronic kidney disease (CKD), end-stage renal disease (ESRD) or death. Pathogenic ANCAs, in particular proteinase 3 (PR3) and myeloperoxidase (MPO), trigger a deleterious immune response resulting in pauci-immune necrotizing and crescentic glomerulonephritis (GN), a common manifestation of glomerular injury in AAV. However, there is growing evidence that activation of the complement pathway contributes to the pathogenesis and progression of AAV. We here aimed to compare glomerular and tubulointerstitial lesions in ANCA GN and extrarenal manifestation of AAV in association with levels of circulating complement components C3c and C4. Methods: Plasma levels of C3c and C4 in a total number of 53 kidney biopsies with ANCA GN were retrospectively included between 2015 and 2020. Glomerular and tubulointerstitial lesions were evaluated according to established scoring systems for ANCA GN and analogous to the Banff classification. Results: We here show that circulating levels of C3c and C4 in ANCA GN were comparable to the majority of other renal pathologies. Furthermore, hypocomplementemia was only detectable in a minor subset of ANCA GN and not correlated with renal or extrarenal AAV manifestations. However, low levels of circulating C3c correlated with AKI severity in ANCA GN independent of systemic disease activity or extrarenal AAV manifestation. By systematic scoring of glomerular and tubulointerstitial lesions, we provide evidence that low levels of circulating C3c and C4 correlated with vasculitis manifestations to distinct renal compartments in ANCA GN. Conclusions: We here expand our current knowledge about distinct complement components in association with vasculitis manifestations to different renal compartments in ANCA GN. While low levels of C4 correlated with glomerulitis, our observation that low levels of circulating complement component C3c is associated with interstitial vasculitis manifestation reflected by intimal arteritis implicates that C3c contributes to tubulointerstitial injury in ANCA GN.

## 1. Introduction

According to the 2012 revised Chapel Hill Consensus Conference Nomenclature of Vasculitides, antineutrophil cytoplasmic antibody (ANCA)-associated vasculitis (AAV) is a small vessel vasculitis, most frequently presenting as microscopic polyangiitis (MPA) or granulomatosis with polyangiitis (GPA) [1,2]. Acute kidney injury (AKI) is a common and severe complication of AAV as it can cause progressive chronic kidney disease (CKD), end-stage renal disease (ESRD) or death [3,4]. Pathogenic ANCAs, in particular proteinase 3 (PR3) and myeloperoxidase (MPO), trigger a deleterious immune response resulting in pauci-immune necrotizing and crescentic glomerulonephritis (GN), a common manifestation of glomerular injury in AAV [5]. Several studies have investigated determinants of renal outcome in ANCA GN, including baseline kidney function and histopathological lesions [6,7]. On a mechanistic level, neutrophils are activated by pathogenic ANCAs causing release of inflammatory cytokines, reactive oxygen species and lytic enzymes, and resulting in excessive formation of neutrophil extracellular traps (NETs) [8,9,10]. At least in part, these mechanisms underly the pathogenicity of ANCAs mediating inflammation and vascular injury. Disease manifestations in the kidney are usually characterized by a pauci-immunity with only minor, if any, immunoglobulin and complement deposition in the vascular system. In general, circulating complement components C3 and C4 levels in AAV are normal [11]. However, there is growing evidence that activation of the complement pathway contributes to the pathogenesis and progression of AAV [12,13,14]. In experimental models, pauci-immune crescentic GN correlated with the activation of the complement system [15]. Furthermore, blocking the alternative complement system resulted in protection from crescentic GN, further supporting an important contribution of the alternative complement system in the pathogenesis of ANCA GN [15]. Conversely, complement C4d has been found in the majority of renal biopsies, implicating that activation of the classical complement system is also relevant in ANCA GN [16]. The mechanistic contribution of the complement system in the pathogenesis of AAV was further substantiated by recent studies that analyzed complement components in different activity states of AAV [16,17,18,19,20,21,22]. This is especially relevant since the use of the anti-C5a receptor inhibitor avacopan has recently been shown to sustain effective remission in AAV, further supporting the importance of the complement system in the pathogenesis and progression of AAV [23,24,25]. However, systematic analysis of the activation of the complement system in association with distinct clinical and histopathological lesions in ANCA GN remains elusive. Therefore, we here aimed to compare glomerular and tubulointerstitial lesions in ANCA GN analogous to the Banff classification and extrarenal manifestation of AAV in association with levels of circulating complement components C3c and C4.

## 2. Results

### 2.1. Description of Demographic and Clinical Characteristics

**Figure 1:**
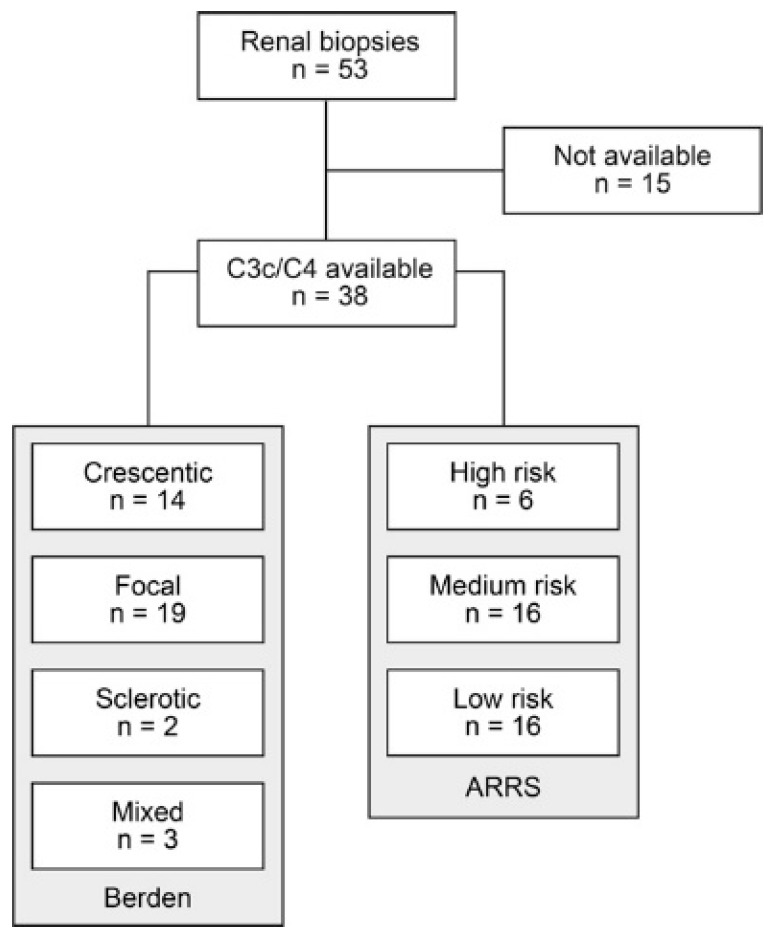
Total patient cohort of ANCA GN. STROBE flow chart of patient disposition with measurements of circulating complement components C3c and C4. Abbreviations: ANCA, anti-neutrophil cytoplasmic antibodies; C3c, complement factor 3 conversion product; C4, complement factor 4; GN, glomerulonephritis; STROBE, Strengthening the Reporting of Observational Studies in Epidemiology.

A total number of 53 renal biopsies with ANCA GN were retrospectively included from 2015 to 2021. Of those, 38 patients with biopsy-proven ANCA GN had available measurements of circulating C3c and C4 levels at disease onset of AAV (Figure 1).

Clinical and laboratory parameters of the total cohort is shown in Table 1. In this cohort, the median (IQR) age at diagnosis was 66.5 (50.75–74) years. All patients were Caucasian and 17/38 (44.7) were female. The median (IQR) disease onset before admission was 17 (7–59) days, kidney biopsy was performed within 6 (3–10) days after admission to confirm renal involvement of AAV. Based on clinical characteristics, 22/38 (57.9%) patients were diagnosed as MPA and the remainder as GPA. A total number of 6/38 (15.8%) patients had a history of vasculitis, the median (IQR) BVAS was 18 (15–20.25). There were 30/38 patients (83%) with extrarenal manifestation of AAV (25 with lung, 5 with sinus, 6 with joint, 2 with ear, 1 with eye, 4 with peripheral nerve and 6 with skin involvement), and 5/38 (13.2%) had alveolar hemorrhage. Based on laboratory findings, there were 22/38 (57.9%) positive for myeloperoxidase (MPO) and 16/38 (42.1%) positive for proteinase 3 (PR3) ANCA. The worst median (IQR) eGFR at disease onset was 17.25 (8.775–47.65) mL/min/1.73 m^2^, and 13/38 (34.2%) required dialysis within 30 days after admission. Median (IQR) levels of circulating C3c were 1.295 g/L (0.9925–1.413 g/L, normal range: 0.82–1.93 g/L) and C4 were 0.26 g/L (0.195–0.3025 g/L, normal range: 0.15–0.57 g/L). Histopathological subgrouping revealed 14/38 (36.8%) crescentic, 19/38 (50%) focal, 2/38 (5.3%) sclerotic and 3/38 (7.9%) mixed class ANCA GN [6]. ARRS was high in 6/38 (15.8%), intermediate in 16/38 (42.1%) and low risk class ANCA GN in 16/38 (42.1%) cases [7].

### 2.2. Hypocomplementemia Is Detectable in a Minor Subset of ANCA GN and Not Correlated with Renal or Extrarenal AAV Manifestations

We first analyzed frequency of hypocomplementemia at disease onset of AAV and separated groups for decreased C3c (<0.82 g/L, normal range: 0.82–1.93 g/L) and C4 levels (<0.15 g/L, normal range: 0.15–0.57 g/L). C3c hypocomplementemia was observed in 5/38 (13.2%) ANCA GN cases, only correlated with decreased lung involvement among all extrarenal AAV manifestations (Table 2). Furthermore, C3c hypocomplementemia was not associated with histopathological subgrouping of ANCA GN or ARRS (Table 2) [6,7].

C4 hypocomplementemia was detectable in 4/38 (10.5%) ANCA GN cases, only correlated with former history of AAV (Table 3). With regard to histopathological findings, C4 hypocomplementemia was not related to any histopathological subgroup of ANCA GN or ARRS (Table 3) [6,7].

**Figure 2:**
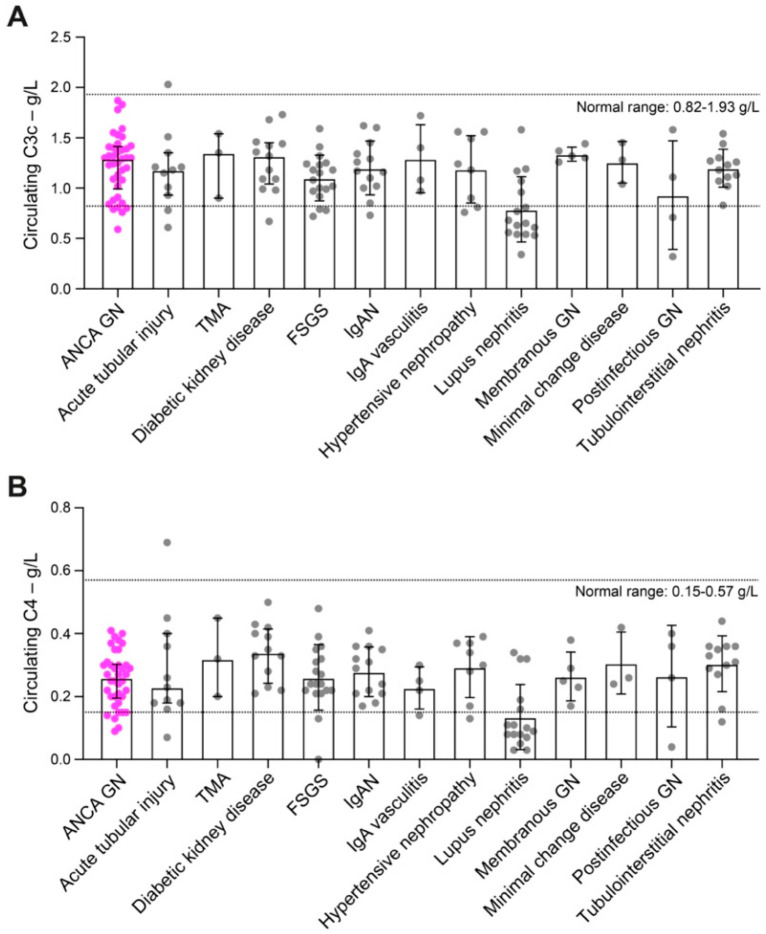
Circulating levels of C3c and C4 in ANCA GN are comparable to the majority of other renal pathologies. (**A**,**B**) The scatter dot plots represent medians and IQR with individual data points summarizing levels of circulating C3c and C4 in ANCA GN (purple dots), acute tubular injury, thrombotic microangiopathy, diabetic kidney disease, FSGS, IgAN, IgA vasculitis, hypertensive nephropathy, lupus nephritis, membranous GN, minimal change disease, postinfectious GN and tubulointerstitial nephritis (all in grey dots). Abbreviations: ANCA, anti-neutrophil cytoplasmic antibodies; C3c, complement factor 3 conversion product; C4, complement factor 4; FSGS, focal segmental glomerulosclerosis; GN, glomerulonephritis; IgAN, IgA nephropathy.

These observations were further substantiated by analysis of circulating complement components in various renal pathologies including acute tubular injury, thrombotic microangiopathy, diabetic kidney disease, focal segmental glomerulosclerosis (FSGS), IgA nephropathy (IgAN), IgA vasculitis, hypertensive nephropathy, lupus nephritis, membranous GN, minimal change disease, postinfectious GN and tubulointerstitial nephritis. Levels of circulating C3c and C4 were comparable to ANCA GN with the exception of lupus nephritis associated with decreased levels of C3c and C4 (Figure 2A,B and Table 4 and Table 5). In summary, circulating levels of C3c and C4 in ANCA GN were comparable to the majority of other renal pathologies. Furthermore, activation of the complement system with associated hypocomplementemia was only present in a minor subset of ANCA GN and not correlated with any renal or extrarenal AAV manifestations.

### 2.3. Low Levels of Circulating C3c Correlates with AKI Severity in ANCA GN Independent of Systemic Disease Activity or Extrarenal AAV Manifestation

**Figure 3:**
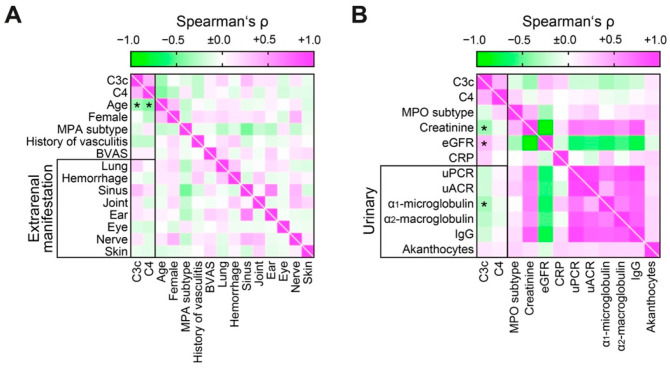
Low levels of circulating C3c correlates with AKI severity in ANCA GN independent of systemic disease activity or extrarenal AAV manifestation. (**A**) Circulating levels of C3c and C4 in association with clinical findings are shown by heatmap reflecting mean values of Spearman’s ρ, asterisks indicate *p* < 0.05. (**B**) Circulating levels of C3c and C4 in association with laboratory parameters are shown by heatmap reflecting mean values of Spearman’s ρ, * asterisks indicate *p* < 0.05. Abbreviations: AAV, ANCA-associated vasculitis; ANCA, anti-neutrophil cytoplasmic antibodies; BVAS, Birmingham Vasculitis Activity Score; C3c, complement factor 3 conversion product; C4, complement factor 4; eGFR, estimated glomerular filtration rate; IgG, immunoglobulin G; MPA, microscopic polyangiitis; MPO, myeloperoxidase; uACR, urinary albumin-to-creatinine ratio; uPCR, urinary protein-to-creatinine ratio.

We next analyzed distinct levels of circulating complement components C3c and C4 with clinical and laboratory parameters in ANCA GN. Levels of C3c and C4 inversely correlated with age, while other clinical findings including ANCA subtype and extrarenal AAV manifestations were not associated with circulating C3c and C4 levels (Figure 3A). Among laboratory parameters, low levels of C3c correlated with AKI severity reflected by rise of serum creatinine and estimated glomerular filtration rate (eGFR) loss (Figure 3B), in line with previous reports (32). Interestingly, low levels of C3c also correlated with tubular proteinuria reflected by α_1_-microglobulin (Figure 3B), implicating that activation of complement component C3c associates with injury to the tubulointerstitial compartment in ANCA GN. In contrast, levels of circulating C4 did not correlate with any aforementioned laboratory parameters (Figure 3B). In summary, low levels of specifically circulating C3c correlated with AKI severity and markers of tubulointerstitial injury in ANCA GN independent of systemic disease activity or extrarenal AAV manifestation.

### 2.4. Low Levels of Circulating C3c and C4 Indicate Vasculitis Manifestations to Distinct Renal Compartments in ANCA GN

**Figure 4:**
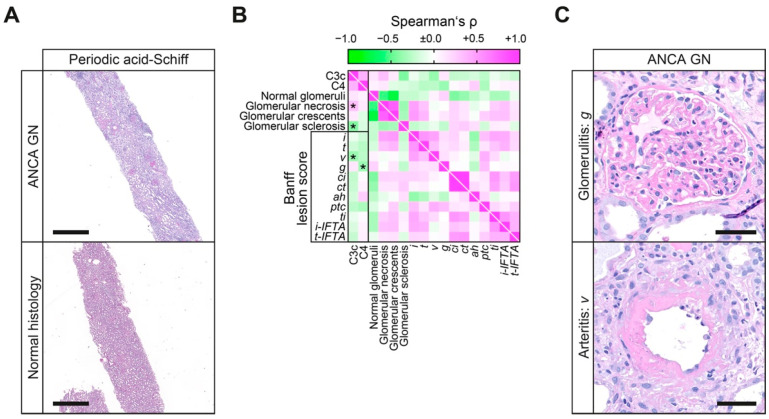
Low levels of circulating C3c and C4 indicate vasculitis manifestations to distinct renal compartments in ANCA GN. (**A**) Representative periodic acid-Schiff-stained kidney section of ANCA GN (scale bar: 1 mm). (**B**) Circulating levels of C3c and C4 in association with glomerular and tubulointerstitial lesions (analogous to the Banff scoring system) are shown by heatmap reflecting mean values of Spearman’s ρ, asterisks indicate *p* < 0.05. (**C**) Representative periodic acid-Schiff-stained kidney section with glomerulitis: *g* and arteritis: *v* lesions in ANCA GN (analogous to the Banff scoring system, scale bars: 50 μm). Abbreviations: *ah*, arteriolar hyalinosis; ANCA, anti-neutrophil cytoplasmic antibodies; C3c, complement factor 3 conversion product; C4, complement factor 4; *ci*, interstitial fibrosis; *ct*, tubular atrophy; *g*, glomerulitis; GN, glomerulonephritis; *i*, interstitial inflammation; *i-IFTA*, inflammation in IFTA; *t*, tubulitis; *ptc*, peritubular capillaritis; *ti*, total inflammation; *t-IFTA*, tubulitis in IFTA; *v*, intimal arteritis.

Based on our previous observation that circulating C3c correlated with AKI and markers of tubulointerstitial injury in ANCA GN, we finally analyzed glomerular and tubulointerstitial lesions in association with complement system components C3c and C4. Levels of C3c were inversely correlated with glomerular necrosis and positively correlated with global glomerular sclerosis (Figure 4A,B). By contrast, no such association with glomerular lesions in ACNA GN was observed for levels of complement component C4 (Figure 4A,B). With regard to tubulointerstitial lesions scored analogous to the Banff classification, complement system activation with low levels of circulating C3c correlated with vasculitis manifestation to the interstitial compartment reflected by intimal arteritis (*v*, Figure 4B,C), further corroborated by multiple regression analysis (Table 6). Conversely, low levels of circulating C4 were associated with vasculitis manifestation to the glomerular compartment reflected by glomerulitis with occlusion of at least one glomerular capillary by leukocyte infiltration and endothelial cell enlargement (*g*, Figure 4B,C). In summary, distinct complement components indicated vasculitis manifestations to different renal compartments in ANCA GN with low levels of C3c associated with interstitial vasculitis and low levels of C4 associated with glomerulitis.

## 3. Discussion

We here show that circulating levels of C3c and C4 in ANCA GN were comparable to the majority of other renal pathologies. Furthermore, hypocomplementemia was only detectable in a minor subset of ANCA GN and not correlated with renal or extrarenal AAV manifestations, in line with previous reports [26]. However, low levels of circulating C3c correlated with AKI severity in ANCA GN independent of systemic disease activity or extrarenal AAV manifestation, again in line with independent reports [27]. By systematic scoring of glomerular and tubulointerstitial lesions in association with circulating complement components C3c and C4, we here provide further insights into the mechanistic role of complement system activation in kidney injury to different renal compartments in ANCA GN. The complement system plays an important role in innate immunity to defend common pathogens [28]. The complement system includes more than 30 plasma and cell surface proteins, amounting to more than 15% of the globular fraction [29]. Activation of the complement system leads to multiple proteolytic cascades, resulting in opsonization and lysis of the pathogen generation of an inflammatory response by the production of potent proinflammatory molecules. The complement system is organized into hierarchical proteolytic cascades initiated by the identification of the pathogenic surface molecules and leading to the generation of potent proinflammatory mediators (anaphylatoxins), opsonization (coating) of the pathogenic surface by various complement opsonins (including complement factor C3), resulting in targeted lysis of the pathogenic surface through the assembly of membrane-penetrating pores known as the membrane attack complex (MAC) [30]. In the context of ANCA GN, minor immunoglobulin and complement deposition has been found in the glomerular capillaries of patients with AAV [26]. However, there is increasing evidence that intrarenal complement component depositions are detectable in a considerable subset of ANCA GN cases, associated with proteinuria and kidney injury [31,32,33]. Although the intensity of complement deposition has been described mild to moderate, complement deposition have been associated with more severe lesions in the kidneys, associated with crescentic ANCA GN and more severe tubulointerstitial lesions [33].

We here expand our current knowledge about distinct complement components in association with vasculitis manifestations to different renal compartments in ANCA GN. In this context, we here show that low levels of C4 correlated with glomerulitis in ANCA GN. The complement system was not considered to be a major contributor in the pathogenesis of AAV, because intrarenal lesions are characterized by an absence or paucity of immune deposits in ANCA GN. However, experimental data from animal models of AAV have suggested that the complement system contributes to the pathogenesis and progression of ANCA GN [12,13]. By transferring MPO-ANCAs into *wildtype* mice or anti-MPO splenocytes into immunodeficient mice, crescentic GN has been observed that could have effectively been blocked by depletion of distinct components of the alternative complement system [15]. Inhibition of complement component C5 by using a monoclonal antibody prevented development of ANCA GN and attenuated crescent formation when administered after disease induction, further supporting that the alternative complement system contributes to the pathogenesis but also progression of ANCA GN [34]. Furthermore, we here provide evidence that low levels of circulating complement component C3c are associated with interstitial vasculitis manifestation reflected by intimal arteritis, implicating that C3c contributes to tubulointerstitial injury. Disease remission of AAV has previously been shown to associate with higher plasma levels of C3a, C5a, soluble C5b-9 and complement factor B as compared to active ANCA GN [18]. Activation of the alternative complement system also correlated with more active systemic disease activity assessed by BVAS, further underscoring the contribution of the alternative complement system to the pathogenesis and progression of ANCA GN and AAV severity [18]. The concept of the complement system contributing to tubulointerstitial injury and inflammation has previously been reported in the context of proteinuria associated with complement activation within the tubulointerstitial compartment [35,36]. Recently, properdin as only positive regulator of the alternative complement system has been shown to contribute to kidney injury due to complement activation by proteinuria [37,38]. Properdin binds to the C3 convertase and partially protects inhibition by factors I and H. In addition, an association between complement activation, tubulointerstitial injury and inflammation has also been reported independent of proteinuria. In experimental models of kidney injury, complement components C3a and C5a have been shown to contribute to tubulointerstitial injury and inflammation [39,40]. Finally, previous studies suggest that the production of C3 by microvascular endothelium of glomerular origin is stimulated by tumor necrosis factor-alpha (TNF)-alpha [41]. Therefore, pro-inflammatory stimuli could mechanistically link complement system activation and AKI in ANCA GN.

The concept of tubulointerstitial injury mediating impairment of kidney function was described more than five decades ago, showing that progressive decline of kidney function exhibited a stronger correlation with the severity of tubulointerstitial rather than with glomerular damage, also described in the context of ANCA GN [42,43,44]. This is further supported by our observation that low levels of C3c correlated with AKI and specifically tubular proteinuria. Tubulointerstitial injury can either follow a glomerular injury or can start directly in the tubulointerstitial compartment as a result of interstitial vasculitis manifestation, including intimal arteritis in ANCA GN [45]. Interestingly, tubulointerstitial nephritis with normal glomeruli in AAV have been described in the first renal biopsy with necrotizing and crescentic ANCA GN in the second renal biopsy thereafter [46,47]. These observations suggest that AAV is a small vessel vasculitis manifesting to glomerular (necrotizing and crescentic ANCA GN) and tubulointerstitial compartments (intimal arteritis), and that the characteristics of each manifestation are independent [48]. Based on our findings that complement components C3 and C4 indicate vasculitis manifestations to distinct renal compartments in ANCA GN, it is attractive to speculated that innate immunity facilitates kidney injury by pathomechanisms attributed to specific complement system components. While C3 is critical for activation of the complement system as a whole, C4 is the major protein of the classical cascade [49]. Anaphylatoxin C3a stimulate inflammation by inducing an oxidative burst in macrophages, eosinophils and neutrophils [50,51,52]. Furthermore, C3a and C5a directly activate basophils and mast cells, resulting in histamine production [53,54]. Even if pro-inflammatory effects of C3a are not in question, studies also highlighted the anti-inflammatory role of C3a in regard to different contexts [55]. Migration of neutrophils and degranulation are prevented in the presence of anaphylatoxin C3a, whether other granulocytes are activated by C3a [56,57]. The C4 activation product C4a has also been shown to have a functional activity on macrophages and monocytes [58,59]. Because no C4a receptor has yet been reported, the physiological role of anaphylatoxin C4a or its contribution to autoimmune diseases including AAV remains elusive [60]. Therefore, more studies are needed to understand the distinct roles of innate immunity, anaphylatoxins C3a and C4a in AAV. However, observed complement system activation in AAV makes targeting specific complement components an attractive therapeutic approach. Currently, two C5a inhibitors are in clinical development for AAV: the oral C5a receptor (C5aR) inhibitor avacopan and the monoclonal C5a antibody IFX-1 [23]. Safety and efficacy with steroid-sparing effects of avacopan in patients with GPA/MPA were shown in Phase II and III clinical trials [23,24,25]. Furthermore, IFX-1 has entered Phase II development [23]. Therefore, measurement of additional circulating complement components (including C5) could be of relevance in association with findings in ANCA GN and therapeutical targeting of the complement system (including anti-C5a receptor inhibition).

The main limitations of our study are its retrospective design and the small patient number. Nevertheless, we here provide a systematic analysis of circulating complement components C3c and C4 in association with distinct clinical and histopathological findings including glomerular and tubulointerstitial lesions in ANCA GN.

## 4. Materials and Methods

### 4.1. Study Population

A total number of 148 kidney biopsies with various intrarenal renal pathologies including 53 cases with ANCA GN at the University Medical Center Göttingen were retrospectively included between 2015 and 2020, the patient cohort has previously been described [45,61,62,63]. While no formal approval was required for the use of routine clinical data, a favorable ethical opinion was granted by the local Ethics committee (protocol codes: 22/2/14, approval date 22 September 2014 and 28/09/17, approval date 17 November 2017). At admission, the Birmingham Vasculitis Activity Score (BVAS) version 3 was assessed [64]. Medical records were used to obtain data on age, sex, duration of disease onset before admission, diagnosis (MPA or GPA) and laboratory results. The estimated glomerular filtration rate (eGFR) was calculated using the Chronic Kidney Disease Epidemiology Collaboration (CKD-EPI) equation [65].

### 4.2. Renal Histopathology

Two renal pathologists (SH and PS) independently evaluated kidney biopsies and were blinded to data analysis. Periodic acid-Schiff stainings were performed by automated slide stainer Tissue-Tek Primsa (Sakura Finetek Europe, Alphen aan den Rijn, Netherlands) according to the manufacturer’s protocol. Within a kidney biopsy, each glomerulus was scored separately for the presence of necrosis, crescents and global sclerosis. Based on these scorings, histopathological subgrouping according to Berden et al. into focal, crescentic, mixed or sclerotic class was performed [6]. Furthermore, the ANCA renal risk score (ARRS) according to Brix et al. into low, medium or high risk was calculated [7]. Kidney biopsies were also evaluated analogous to the Banff scoring system for allograft pathology as described previously [66]. In brief, Banff score lesions included interstitial inflammation (*i*), tubulitis (*t*), arteritis (*v*), glomerulitis (*g*), interstitial fibrosis (*ci*), tubular atrophy (*ct*), arteriolar hyalinosis (*ah*), peritubular capillaritis (*ptc*), total inflammation (*ti*), inflammation in areas of IFTA (*i-IFTA*) and tubulitis in areas of IFTA (*t-IFTA*) [66].

### 4.3. C3c and C4 Measurements

Plasma concentrations of human complement components C3c (9D9621, Abbott, Chicago, IL, USA) and C4 (9D9721, Abbott, Chicago, IL, USA) were determined by turbidimetric measurements on the ARCHITECT-C module. Normal range plasma concentrations for circulating C3c is defined between 0.82–1.93 g/L and C4 between 0.15–0.57 g/L.

### 4.4. Statistical Methods

Variables were tested for normal distribution using the Shapiro-Wilk test. Statistical comparisons were not formally powered or prespecified. Non-normally distributed continuous variables are shown as median and interquartile range (IQR), categorical variables are presented as frequency and percentage. For group comparisons, the Mann-Whitney U-test was used to determine differences in medians. Non-parametric between-group-comparisons were performed with Pearson’s Chi-square test. Univariate analyses were performed by nonparametric Spearman correlation. Data analyses were performed with GraphPad Prism (version 8.4.3 for MacOS, GraphPad Software, San Diego, CA, USA). Multiple regression analyses were performed using IBM SPSS Statistics (version 27 for MacOS, IBM Corporation, Armonk, NY, USA). We retained covariates significantly associated with complement component measurements in a multivariable regression model, limiting the model covariates to avoid model over-fit. A probability (*p*) value of <0.05 was considered statistically significant.

## 5. Conclusions

We here expand our current knowledge about distinct complement components in association with vasculitis manifestations to different renal compartments in ANCA GN. While low levels of C4 correlated with glomerulitis, our observation that low levels of circulating complement component C3c is associated with interstitial vasculitis manifestation reflected by intimal arteritis implicates that C3c contributes to tubulointerstitial injury in ANCA GN.

## Figures and Tables

**Table 1:** Clinical and laboratory parameters of the total ANCA GN cohort.

Parameter	Value
Median age (IQR)—years	66.5 (50.75–74)
Female sex—no. (%)	17 (44.7)
Disease onset—days before admission (IQR)	17 (7–59)
Kidney biopsy—days after admission (IQR)	6 (3–10)
ANCA subtype MPA/GPA—no. (%)	22 (57.9)
History of vasculitis—no. (%)	6 (15.8)
Dialysis within 30 days after admission—no. (%)	13 (34.2)
Median BVAS (IQR)—points	18 (15–20.25)
Extrarenal manifestation—no. (%)	30 (78.9)
Lung involvement—no. (%)	25 (65.8)
Pulmonary hemorrhage—no. (%)	5 (13.2)
Sinus involvement—no. (%)	5 (13.2)
Joint involvement—no. (%)	6 (15.8)
Ear involvement—no. (%)	2 (5.3)
Eye involvement—no. (%)	1 (2.6)
Nerve involvement—no. (%)	4 (10.5)
Skin involvement—no. (%)	6 (15.8)
Median serum creatinine (IQR)—mg/dL	22 (57.9)
ANCA subtype MPO/PR3—no. (%)	3.155 (1.475–5.215)
Median eGFR (IQR)—mL/min/1.73 m^2^	17.25 (8.775–47.65)
Median CRP (IQR)—mg/L	60.5 (19.5–101.2)
C3c (IQR)—g/L	1.295 (0.9925–1.413)
C4 (IQR)—g/L	0.26 (0.195–0.3025)
Median uPCR (IQR)—mg/g	1027 (508.9–1841)
Median uACR (IQR)—mg/g	430.8 (211.2–897.2)
Median α_1_-microglobulin (IQR)—mg/g	83.17 (33.2–187.8)
Median α_2_-macroglobulin (IQR)—mg/g	5.14 (2.935–12.25)
Median IgG (IQR)—mg/g	52.88 (23.3–205.5)
Acanthocytes—no. (%)	6 (15.8)
Crescentic class—no. (%)	14 (36.8)
Focal class—no. (%)	19 (50)
Sclerotic class—no. (%)	2 (5.3)
Mixed class—no. (%)	3 (7.9)
ARRS high risk—no. (%)	6 (15.8)
ARRS medium risk—no. (%)	16 (42.1)
ARRS low risk—no. (%)	16 (42.1)

Abbreviations: ANCA, anti-neutrophil cytoplasmic antibodies; ARRS, ANCA renal risk score; BVAS, Birmingham Vasculitis Activity Score; C3c, complement factor 3 conversion product; C4, complement factor 4; CRP, C-reactive protein; eGFR, estimated glomerular filtration rate (CKD-EPI); GN, glomerulonephritis; GPA, granulomatosis with polyangiitis; IQR, interquartile range; MPA, microscopic polyangiitis; MPO, myeloperoxidase; No., number; PR3, proteinase 3; uPCR, urinary protein-to-creatinine ratio; uACR, urinary albumin-to-creatinine ratio.

**Table 2:** Baseline patient characteristics according to circulating C3c levels (normal range: 0.82–1.93 g/L).

Parameter	Decreased C3c	Normal C3c	*p* Value
No. of patients	5	33	
Median age (IQR)—years	76 (55–76.5)	66 (50.5–72.5)	0.2655
Female sex—no. (%)	2 (40)	15 (45.5)	0.8192
ANCA subtype MPA/GPA—no. (%)	3 (60)	19 (57.6)	0.9185
History of vasculitis—no. (%)	2 (40)	4 (12.1)	0.1111
Median BVAS (IQR)—points	18 (12–22.5)	18 (15.5–20.5)	0.7950
Lung involvement—no. (%)	1 (20)	24 (72.7)	**0.0206**
Pulmonary hemorrhage—no. (%)	0 (0)	5 (15.2)	0.3503
Sinus involvement—no. (%)	0 (0)	5 (15.2)	0.3503
Joint involvement—no. (%)	0 (0)	6 (18.2)	0.2988
Ear involvement—no. (%)	0 (0)	2 (6.1)	0.5717
Eye involvement—no. (%)	0 (0)	1 (3)	0.6932
Nerve involvement—no. (%)	0 (0)	4 (12.1)	0.4105
Skin involvement—no. (%)	0 (0)	6 (18.2)	0.2988
ANCA subtype MPO/PR3—no. (%)	3 (60)	19 (57.6)	0.9185
Median serum creatinine (IQR)—mg/dL	3.74 (2.27–6.235)	3.14 (1.385–5.095)	0.4777
Median eGFR (IQR)—mL/min/1.73 m^2^	16.9 (8.9–22.75)	21.4 (8.65–50.25)	0.5252
Median CRP (IQR)—mg/L	57.4 (30.05–112.2)	63.6 (19.1–96.3)	0.7337
Median C4 (IQR)—g/L	0.17 (0.115–0.295)	0.27 (0.205–0.305)	0.1087
Median uPCR (IQR)—mg/g	556.9 (428.6–5860)	1157 (550.7–1641)	0.9999
Median uACR (IQR)—mg/g	362.3 (81.12–2644)	445.2 (231.6–854.6)	0.9999
Median α_1_-microglobulin (IQR)—mg/g	125.9 (61.9–258.7)	69.7 (31.6–173.8)	0.2707
Median α_2_-macroglobulin (IQR)—mg/g	7.38 (4.325–27.03)	5.14 (2.868–12.25)	0.4211
Median IgG (IQR)—mg/g	149 (9.555–259.1)	48.61 (25.85–190.8)	0.8464
Acanthocytes—no. (%)	0 (0)	6 (18.2)	0.2988
Crescentic class—no. (%)	1 (20)	13 (39.4)	
Focal class—no. (%)	3 (60)	16 (48.5)	
Sclerotic class—no. (%)	1 (20)	1 (3)	
Mixed class—no. (%)	0 (0)	3 (9.1)	0.3355
ARRS high risk—no. (%)	1 (20)	5 (15.2)	
ARRS medium risk—no. (%)	2 (40)	14 (42.4)	
ARRS low risk—no. (%)	2 (40)	14 (42.4)	0.9623

For group comparisons, the Mann-Whitney U-test was used to determine differences between medians. Non-parametric between-group-comparisons were performed with Pearson’s Chi-square test. Bold indicates statistically significant values at group level. Abbreviations: ANCA, anti-neutrophil cytoplasmic antibodies; ARRS, ANCA renal risk score; BVAS, Birmingham Vasculitis Activity Score; C3c, complement factor 3 conversion product; C4, complement factor 4; CRP, C-reactive protein; eGFR, estimated glomerular filtration rate (CKD-EPI); GN, glomerulonephritis; GPA, granulomatosis with polyangiitis; IQR, interquartile range; MPA, microscopic polyangiitis; MPO, myeloperoxidase; No., number; PR3, proteinase 3; uPCR, urinary protein-to-creatinine ratio; uACR, urinary albumin-to-creatinine ratio.

**Table 3:** Baseline patient characteristics according to circulating C4 levels (normal range: 0.15–0.57 g/L).

Parameter	Decreased C4	Normal C4	*p* Value
No. of patients	5	33	
Median age (IQR)—years	65 (46.75–74.25)	66.5 (50.75–74)	0.9358
Female sex—no. (%)	3 (75)	14 (41.2)	0.1981
ANCA subtype MPA/GPA—no. (%)	2 (50)	20 (58.8)	0.7353
History of vasculitis—no. (%)	2 (50)	4 (11.8)	**0.0473**
Median BVAS (IQR)—points	19 (13.75–19.75)	18 (15–21)	0.8940
Lung involvement—no. (%)	3 (75)	22 (64.7)	0.6914
Pulmonary hemorrhage—no. (%)	1 (25)	4 (11.8)	0.4589
Sinus involvement—no. (%)	0 (0)	5 (14.7)	0.4105
Joint involvement—no. (%)	1 (25)	5 (14.7)	0.5933
Ear involvement—no. (%)	0 (0)	2 (5.9)	0.6182
Eye involvement—no. (%)	0 (0)	1 (2.9)	0.7281
Nerve involvement—no. (%)	0 (0)	4 (11.8)	0.4683
Skin involvement—no. (%)	1 (25)	5 (14.7)	0.4105
ANCA subtype MPO/PR3—no. (%)	1 (25)	21 (61.8)	0.1589
Median serum creatinine (IQR)—mg/dL	2.365 (1.798–2.978)	3.49 (1.423–5.538)	0.4788
Median eGFR (IQR)—mL/min/1.73 m^2^	25 (19.23–27.4)	15.55 (8.4–49.33)	0.5322
Median CRP (IQR)—mg/L	41.8 (14.65–160.2)	63.7 (19.5–101.2)	0.8687
Median C3c (IQR)—g/L	0.8 (0.6325–1.613)	1.3 (1.088–1.413)	0.1506
Median uPCR (IQR)—mg/g	920.1 (408.7–1407)	1027 (508.9–2222)	0.5351
Median uACR (IQR)—mg/g	560.9 (129–831.8)	430.8 (211.2–1028)	0.8725
Median α_1_-microglobulin (IQR)—mg/g	53.45 (24.07–95.5)	88 (33.2–194.5)	0.3680
Median α_2_-macroglobulin (IQR)—mg/g	4.94 (2.92–7.61)	5.22 (2.915–13.37)	0.7050
Median IgG (IQR)—mg/g	22.71 (6.563–84.23)	57.85 (26.74–214.3)	0.1267
Acanthocytes—no. (%)	0 (0)	6 (17.6)	0.3599
Crescentic class—no. (%)	2 (50)	12 (35.3)	
Focal class—no. (%)	2 (50)	17 (50)	
Sclerotic class—no. (%)	0 (0)	2 (5.9)	
Mixed class—no. (%)	0 (0)	3 (8.8)	0.8499
ARRS high risk—no. (%)	0 (0)	6 (17.6)	
ARRS medium risk—no. (%)	1 (25)	15 (44.1)	
ARRS low risk—no. (%)	3 (75)	13 (38.2)	0.3387

For group comparisons, the Mann-Whitney U-test was used to determine differences between medians. Non-parametric between-group-comparisons were performed with Pearson’s Chi-square test. Bold indicates statistically significant values at group level. Abbreviations: ANCA, anti-neutrophil cytoplasmic antibodies; BVAS, Birmingham Vasculitis Activity Score; C3c, complement factor 3 conversion product; C4, complement factor 4; CRP, C-reactive protein; eGFR, estimated glomerular filtration rate (CKD-EPI); GN, glomerulonephritis; GPA, granulomatosis with polyangiitis; IQR, interquartile range; MPA, microscopic polyangiitis; MPO, myeloperoxidase; No., number; PR3, proteinase 3; uPCR, urinary protein-to-creatinine ratio; uACR, urinary albumin-to-creatinine ratio.

**Table 4:** Levels of circulating C3c among different renal pathologies (normal range: 0.82–1.93 g/L).

Renal Pathology	No. of Patients	Median C3c (IQR)—g/L	*p* Value vs. ANCA GN
ANCA GN	38	1.295 (0.9925–1.413)	0.9999
Acute tubular injury	11	1.18 (0.93–1.35)	0.9999
Thrombotic microangiopathy	3	1.35 (0.9–1.54)	0.9999
Diabetic kidney disease	13	1.32 (1.04–1.45)	0.9999
FSGS	18	1.115 (0.9575–1.243)	0.9999
IgAN	13	1.17 (1.01–1.395)	0.9999
IgA vasculitis	4	1.24 (0.9975–1.64)	0.9999
Hypertensive nephropathy	8	1.21 (0.8325–1.543)	0.9999
Lupus nephritis	16	0.665 (0.545–1.043)	**0.0002**
Membranous GN	5	0.32 (1.28–1.405)	0.9999
Minimal change disease	3	1.28 (1.04–1.45)	0.9999
Postinfectious GN	4	0.91 (0.4175–1.463)	0.9999
Tubulointerstitial nephritis	12	1.205 (1.08–1.293)	0.9999

For group comparisons, the Kruskal-Wallis test was used for multiple comparisons. Bold indicates statistically significant values at group level. Abbreviations: ANCA, anti-neutrophil cytoplasmic antibodies; C3c, complement factor 3 conversion product; FSGS, focal segmental glomerulosclerosis; GN, glomerulonephritis; IgA, immunoglobulin A; IgAN, IgA nephropathy.

**Table 5:** Levels of circulating C4 among different renal pathologies (normal range: 0.15–0.57 g/L).

Renal Pathology	No. of Patients	Median C4 (IQR)—g/L	*p* Value vs. ANCA GN
ANCA GN	38	0.26 (0.195–0.2035)	0.9999
Acute tubular injury	11	0.23 (0.18–0.4)	0.9999
Thrombotic microangiopathy	3	0.32 (0.2–0.45)	0.9999
Diabetic kidney disease	13	0.34 (0.2425–0.415)	0.2124
FSGS	18	0.24 (0.2175–0.3275)	0.9999
IgAN	13	0.29 (0.21–0.355)	0.9999
IgA vasculitis	4	0.235 (0.16–0.2875)	0.9999
Hypertensive nephropathy	8	0.32 (0.1975–0.37)	0.9999
Lupus nephritis	16	0.095 (0.0725–0.1825)	**0.0145**
Membranous GN	5	0.25 (0.2–0.335)	0.9999
Minimal change disease	3	0.26 (0.24–0.42)	0.9999
Postinfectious GN	4	0.31 (0.095–0.39)	0.9999
Tubulointerstitial nephritis	12	0.32 (0.275–0.36)	0.9999

For group comparisons, the Kruskal-Wallis test was used for multiple comparisons. Bold indicates statistically significant values at group level. Abbreviations: ANCA, anti-neutrophil cytoplasmic antibodies; C4, complement factor 4; FSGS, focal segmental glomerulosclerosis; GN, glomerulonephritis; IgA, immunoglobulin A; IgAN, IgA nephropathy.

**Table 6:** Multiple regression analysis for variables associated with circulating C3c.

Parameter	ß	SE	*p* Value
Glomerular necrosis—%	0.2611	0.1528	0.1208
Glomerular sclerosis—%	−0.3401	0.2093	**0.0485**
Arteritis: *v*—lesion score	−0.3974	0.0425	**0.0171**

Bold indicates statistically significant values. Abbreviations: C3c, complement factor 3 conversion product; SE, standard error.

## Data Availability

Deidentified data are available on reasonable request from the corresponding author.
